# A Silver Nanocluster Assembled by a Superatomic Building Unit

**DOI:** 10.1021/acs.inorgchem.4c00139

**Published:** 2024-03-12

**Authors:** Wei-Jung Yen, Jian-Hong Liao, Tzu-Hao Chiu, Yuh-Sheng Wen, C. W. Liu

**Affiliations:** † Department of Chemistry, National Dong Hwa University, Hualien 97401, Taiwan, Republic of China; ‡ Institute of Chemistry, Academia Sinica, Taipei 11528, Taiwan, Republic of China

## Abstract

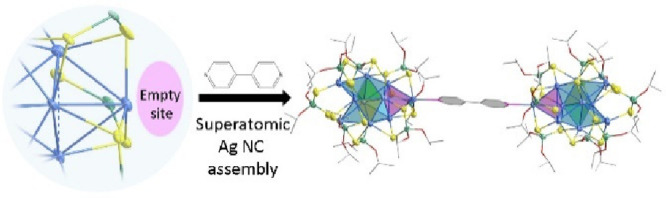

A unique assembly of a two-electron superatom, [Ag_10_{S_2_P(O^*i*^Pr)_2_}_8_], as a primary building unit in the construction of a supramolecule [Ag_10_{S_2_P(O^*i*^Pr)_2_}_8_]_2_(μ-4,4′-bpy) through a 4,4′-bipyridine (4,4′-bpy) linker is reported. This approach is facilitated by an open site in the structure that allows for effective pairing. The assembled structure demonstrates a minimal solvatochromic shift across organic solvents with variable polarities, highlighting the influence of self-assembly on the photophysical properties of silver nanoclusters.

## Abstract 2

Utilizing a two-electron superatom, [Ag_10_(dtp)_8_] (dtp = dithiophosphate), as a building block, this study engineered a novel assembly through organic linkers to yield [Ag_10_(dtp)_8_]_2_(μ-4,4′-bpy) (4,4′-bpy = 4,4′-bipyridine). Highlighting the influence of solvent polarity on optical behavior, this work creates a channel to fine-tune the photophysical characteristics of silver nanoclusters.

Metal nanoclusters (NCs) have gained significant attention due to their remarkable electronic, optical, magnetic, and catalytic properties,^1−4^ which are intricately influenced by factors such as size, composition, and surface structure. To further unlock the potential of these NCs, researchers have turned to the concept of self-assembly.^5−11^ By the harnessing of self-assembly principles, it becomes possible to guide the organization of metal NCs into larger, well-ordered structures. Cluster assembly encompasses the intricate arrangement of metal atoms or small molecular units, leading to the formation of larger nanoscale clusters. This process is governed by the interactions between the clusters, wherein surface ligands assume a pivotal role that possesses diverse functionalities to facilitate intercluster interactions, including π···π, C–H···π, electrostatic, and metallophilic interactions.^12−15^ However, these intermolecular interactions exhibit relatively weak strength. In contrast, implementing organic linkers in the nanomolecular assembly has emerged as a promising strategy. In the regime of silver cluster-assembled materials (SCAMs), linkers such as pyrazine,^16^ 1,4-bis(4-pyridyl)benzene,^16^ dipyridin-4-yldiazene (dpd),^16^ 4,4′-bipyridine (bpy),^16−18,24^ 1,2-bis(4-pyridyl)acetylene,^19^ 1,4-bis(pyridin-4-ylethynyl)benzene (bpeb),^19^ 3-amino-4,4′-bipyridine,^20^ pyridinecarboxylic hydrazide (*o*-, *m*-, and *p*-iah),^21^*trans*-1,2-bis(4-pyridyl)ethylene,^22^ 5,10,15,20-tetra(4-pyridyl)porphyrin,^23^ 2,2′,7,7′-tetra(pyridin-4-yl)-9,9′-spirobi(fluorene),^11^ and (2-thiazolyl)sulfide^25,26^ have been employed as more robust and more stable linkages, providing enhanced control over the assembly process. Notably, SCAMs offer many advantages that significantly enhance the functionality, stability, and applicability. These benefits stem from the versatile and tunable nature of organic linkers, which can be engineered to impart specific properties to cluster assemblies. By enhancement of the structural diversity and complexity, these linkers enable the creation of materials with tailored geometries and dimensionalities. Organic linkers also contribute to the improved stability of SCAMs, protecting metal clusters from environmental degradation and extending their functional lifespan. A noteworthy example is the work by Negishi et al., who utilized a 3D silver(I) cluster-assembled material as a surface-enhanced Raman scattering sensor for the detection of Hg^2+^ ions.^19^ This innovation marks a significant expansion in the application domains of SCAMs, demonstrating their potential beyond traditional uses. Despite these advancements, research in this field is still in its nascent stages, indicating a vast scope for exploration and discovery in utilizing SCAMs for environmental monitoring and beyond.

In general, SCAMs typically feature a secondary building unit (SBU) composed of Ag^I^ atoms, which possess zero electrons. However, a notable exception was reported by Mak et al. in 2018,^16^ following their establishment of the first 2D SCAM, [Ag_12_(S^*t*^Bu)_8_(CF_3_COO)_4_(bpy)_4_]_*n*_, in 2017.^18^ In this exceptional case, a two-electron [Ag_14_(C_2_B_10_H_10_S_2_)_6_]^0^ NC was utilized as the cluster node, which was connected by pyrazine, dpd, bpy, and bpeb ligands, resulting in formation from 1D to 3D frameworks. This discovery marked the first instance of Ag^0^-containing superatomic NCs being employed as SBUs in the construction of SCAMs. The Ag_14_ skeleton is composed of an [Ag_6_]^4+^ octahedron with eight face-capping Ag^I^ atoms. The linker ligands connect to these face-capping Ag^I^ atoms, thereby constructing polymeric species. In any case, investigations on a superatomic Ag NC as a SBU remain underexplored. In our previous study, we reported an ultrasmall two-electron Ag NC, [Ag_10_{S_2_P(O^*i*^Pr)_2_}_8_] (denoted as **Ag**_**10**_).^27^ Interestingly, the presence of an external Ag atom with an unoccupied coordination site suggested the potential for subsequent reactions. Building upon this observation, the current investigation employed bpy as the linker to connect two **Ag**_**10**_ NCs, resulting in the formation of [Ag_10_{S_2_P(O^*i*^Pr)_2_}_8_]_2_(μ-bpy) (denoted as **Ag**_**10**_**bpy**). Notably, the **Ag_10_** NCs, once assembled, exhibited photoluminescence (PL) within the near-infrared (NIR-I) region at ambient temperature in solution along with an elevated QY. This underscores their promising utility in applications such as bioimaging and biosensing.^28,29^

**Ag**_**10**_**bpy** was synthesized by mixing **Ag**_**10**_ and bpy ligands in a tetrahydrofuran (THF) solution with a molar ratio of 1:10. The solution was allowed to stand for 1 week to collect crystals as the product. The resulting yield of crystalline products is ca. 85%. It should be noted that decreasing the portion of linkers in the reaction adversely affected the crystal yield. We employed a shorter linker, e.g., pyrazine, in the reaction. Nevertheless, we have not succeeded in obtaining crystals. This challenge may be attributed to the steric hindrance caused by the intermolecular dithiophosphate (dtp) ligands associated with the short contact. In contrast to the previous studies,^16,17,19,20,23^ which utilized a Ag^I^-L (L = thiolate/acetate) complex as a precursor in the assembly reaction, *our approach directly employs two-electron superatom in the synthesis*. This methodology facilitates a more precise and controlled synthesis while potentially mitigating the formation of excessive byproducts.

The ^31^P{^1^H} NMR spectrum of **Ag**_**10**_**bpy** (Figure S1) shows a prominent resonance at 103.3 ppm and two resonances at 104.4 and 105.1 ppm, suggesting the presence of several coordination modes. Given the proximity of the three signals within the spectrum, the integration reveals that their area ratios closely approximate a 2:1:5 distribution. These ratios directly correlate to the distinct coordination modes exhibited by the ligand as follows: (μ_2_, μ_1_), P2 and P6; (μ_1_, μ_1_), P7; (μ_2_, μ_2_), P1, P3, P4, P5, and P8.

The crystal structure of **Ag**_**10**_**bpy** shows a pair of [Ag_10_{S_2_P(O^*i*^Pr)_2_}_8_] molecules connected by bpy as a linker through the external Ag atom (Figure 1a). It crystallized at space group *P*1̅ and showed two half-molecules (clusters **I** and **II**) in the asymmetric unit. Because the bond distances in the two molecules are very similar, only the distance of cluster **I** will be mentioned below. Relevant distances are summarized in Table S2. The entire molecule possesses *C*_*i*_ symmetry, where the inversion center is located at the center of the bpy linker, equally divided into two six-membered rings. The Ag_10_ framework in **Ag**_**10**_**bpy** retains the geometry of a tetracapped trigonal bipyramid and an extended capping Ag_ext_ (Figure 1b). The Ag···Ag distances in the two tetrahedra of the bipyramid are similar [avg. 2.8932(9) Å in yellow Td; avg. 2.8775(9) Å in green Td], while that in the capping tetrahedra is slightly longer [avg. 2.9567(9) Å in cyan Td]. In comparison to **Ag**_**10**_, the average Ag···Ag distance within each tetrahedron in the metal framework is marginally shorter [2.8562(13) Å in yellow Td, 2.8553(13) Å in green Td, and 2.9495(13) Å in cyan Td], indicating a more pronounced argentophilic interaction. Notably, an empty site was observed within the Ag_ext_S_3_ motif in **Ag**_**10**_ (Figure 1c). This unique vacancy suggests the potential introduction of organic solvents or heteroligands to this specific position. The connection of the bpy linker leads to an elongation between Ag_ext_ and its trigonal bottom (2.593 Å in **Ag**_**10**_**bpy**; 2.353 Å in **Ag**_**10**_), resulting in a unique μ_4_-Ag_ext_ in a pyramidal geometry (Figure 1d), thus offering a fixed distance between two **Ag**_**10**_ motifs. The N–Ag_ext_ and Ag_ext_···Ag_ext_ distances in **Ag**_**10**_**bpy** are 2.395(7) and 11.871(1) Å, respectively. The coordination modes of the dtp ligands in **Ag**_**10**_**bpy** (Figure 1c) are consistent with those in **Ag**_**10**_ (Figure 1e). Specifically, the ligands on P1, P3, P4, and P5 maintain a tetrametallic tetraconnective (η^4^:μ_2_, μ_2_) mode and that on P8 is in a trimetallic tetraconnective (η^3^:μ_2_, μ_2_) mode, while those on P2 and P6 adopt a trimetallic triconnective (η^3^:μ_1_, μ_2_) mode and that on P7 is in a bimetallic diconnective (η^2^:μ_1_, μ_1_) mode. The sum of the rotation angles in the Ag_ext_S_3_ motif in both **Ag**_**10**_ (Figure 1f) and **Ag**_**10**_**bpy** (Figure 1g) reveals noteworthy distinctions. The former case shows that the cumulative angle closely approximates 360°, indicative of the Ag_ext_S_3_ motif’s close alignment with a coplanar arrangement, thereby facilitating a solvent molecule proximity. Conversely, in **Ag**_**10**_**bpy**, the introduction of bpy ligands results in a pyramidal geometry.

**Figure 1:**
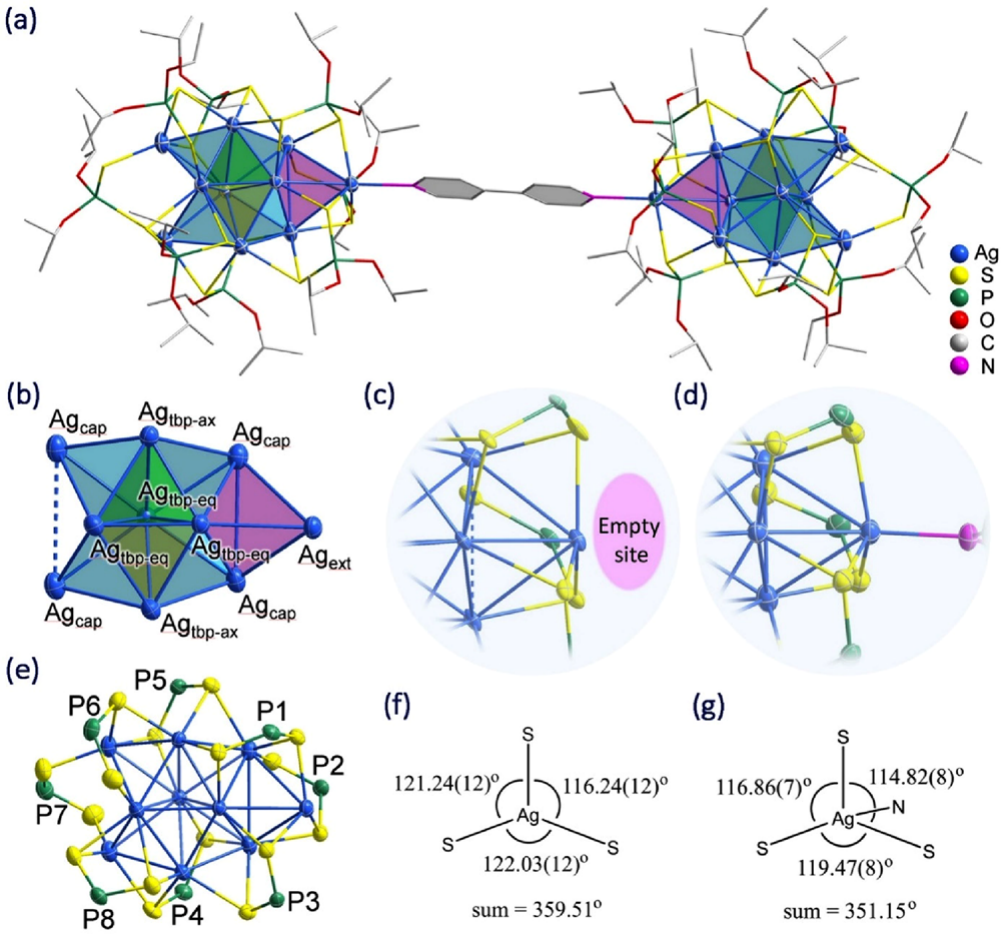
(a) Total structure of **Ag**_**10**_**bpy**. (b) Ag_10_ skeleton in **Ag**_**10**_**bpy**. (c) Enlarged view of the area near the Ag_ext_S_3_ motif in **Ag**_**10**_ (d) and **Ag**_**10**_**bpy**. (e) Ag_10_{S_2_P(O^*i*^Pr)_2_}_8_ motif in **Ag**_**10**_**bpy** (the isopropoxy groups and bpy were omitted for clarity). (f) Sum of the rotation angle at the Ag_ext_S_3_ motif in **Ag**_**10**_ and (g) **Ag**_**10**_**bpy**. Thermal ellipsoids were drawn at 30% probability.

The absorption spectrum of **Ag**_**10**_**bpy** exhibits two prominent bands at 389 and 516 nm, accompanied by a shoulder at 347 nm (Figure 2a). This pattern bears similarity to that of the previously reported **Ag**_**10**_ (348, 392, and 520 nm).^27^ The emission maximum of **Ag**_**10**_**bpy** at 749 nm closely resembles that of **Ag**_**10**_. The predominant portion of the emitted light range is situated within the NIR-I region. Despite this similarity, there is a slight reduction in the quantum yield (QY) for **Ag**_**10**_**bpy**, which stands at 2.3%, in contrast to discrete **Ag**_**10**_ with a QY of 6%. This reduction might be attributed to the linker in **Ag**_**10**_**bpy**, which connects the Ag_ext_ atoms, increasing the distance between Ag_ext_ and nearby Ag atoms by about 0.2 Å. This increased distance, averaging 3.202 Å in **Ag**_**10**_**bpy** compared to 3.022 Å in **Ag**_**10**_, could lead to decreased argentophilicity, promoting energy release through nonradiative vibrational relaxation pathways. Additionally, a temperature-dependent blue shift of 63 nm is observed in **Ag**_**10**_**bpy** when the temperature is lowered from 298 to 77 K. The PL decay curve for **Ag**_**10**_**bpy** aligns well with a single-exponential fitting curve. The emission lifetime of **Ag**_**10**_**bpy** is 2.4 ns at room temperature (RT; Figure S4) and 14.5 ns at 77 K (Figure S5), exhibiting the fluorescence origin of the emission. The photophysical data are summarized in Table S3. Overall, the absorption and emission spectra exhibit consistency after the assembly of **Ag**_**10**_ NCs, showing subtle shifts that uphold the electronic characteristics of the two-electron superatoms.

**Figure 2:**
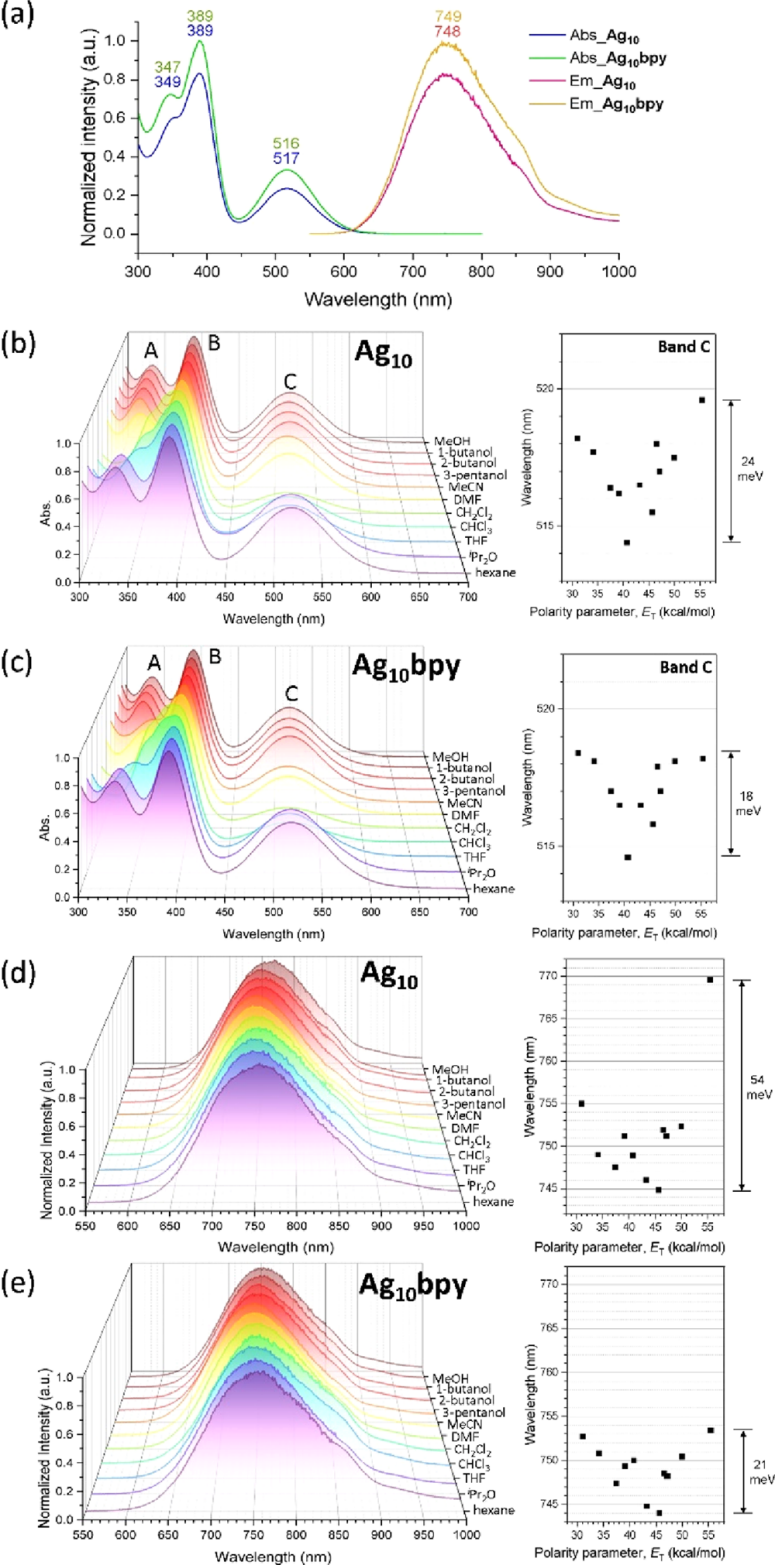
(a) Absorption and emission spectra of **Ag**_**10**_ and **Ag**_**10**_**bpy** in THF at RT. (b) Solvent-dependent absorption spectra of **Ag**_**10**_ and (c) **Ag**_**10**_**bpy** at RT. (d) Solvent-dependent emission spectra of **Ag**_**10**_ and (e) **Ag**_**10**_**bpy** at RT.

Following its assembly with linker ligands, the **Ag**_**10**_**bpy** molecule experiences a discernible change in its molecular shape. This structural modification potentially gives rise to an alteration in the molecular dipole moment.^32^ To evaluate this hypothesis, we conducted a solvent-dependent absorption spectral analysis. The solvent-dependent UV–vis spectra (Figure 2b,c) show discernible shifts in the absorption bands when dissolved in solvents with varying polarities. The polarity of the solvents (Figure S6) is quantified using the polarity parameter (*E*_T_), which is defined by the molar transition energy (measured in kilocalories per mole).^33^ Band A exhibits a heightened sensitivity to variations in the solvent polarity. This behavior is logically consistent with its classification as a part of the ligand-to-metal charge-transfer band, particularly due to the ligands being situated at the outermost layer and thus being more prone to interact with solvent molecules. On the other hand, bands B and C initially exhibit blue-shifting and then red-shifting with increasing solvent polarity. The observation of a blue shift as the solvent polarity increases aligns with the behavior exhibited by the eight-electron NCs Au_22–*x*_Ag_*x*_Cd_1_(SAdm)_15_X (*x* ∼ 3; X = Br/Cl), Au_22_Cd_1_(SAdm)_15_Br, and Au_19_Ag_4_(SAdm)_15_.^32^ It is noted that the previous study did not employ a solvent of higher polarity, with the most polar solvent used being CH_2_Cl_2_. Band C primarily involves a 1S → 1P_*x*_ transition, wherein the orientation of its 1P_*x*_ orbital is oriented toward Ag_ext_, rendering it susceptible to influences from bpy ligands or solvents attached to this site. Band C displays a slight shift (∼18 meV) in **Ag**_**10**_**bpy**, whereas **Ag**_**10**_ exhibits more shifts (24 meV). In contrast, other NCs characterized by low dipole moments (μ < 4 D), such as Au_30_(S^*t*^Bu)_18_,^34^ [Au_25_(SC_2_H_4_Ph)_18_]^−^,^35^ [Au_25_(SC_2_H_4_Ph)_18_]^0^,^36^ and Au_21_(SAdm)_15_,^37^ show smaller peak shifts (<14 meV).^28^ Our observations suggest that the dipole moment of **Ag**_**10**_ is higher than that of **Ag**_**10**_**bpy**. In essence, merging separate entities with their own distinct dipole moments can lead to a new structure in which these moments partially cancel each other out. The assembly of **Ag**_**10**_ (*C*_1_) into **Ag**_**10**_**bpy** (*C*_*i*_) results in a more symmetrical molecular shape, leading to a reduction in the molecule’s dipole moment and a consequent decrease in its susceptibility to solvent polarity.

The solvent-dependent emission spectra reveal a consistent trend, wherein **Ag**_**10**_ exhibits a more pronounced peak shift of 54 meV compared to that of **Ag**_**10**_**bpy** (21 meV). In addition to reducing the dipole moment after assembly, another reason may be that the linker blocks the open site on Ag_ext_, avoiding the interaction of various solvent molecules with this site. The emission in **Ag**_**10**_ originates from the transition of 1P_*x*_ to 1S. Consequently, solvent molecules can significantly influence **Ag**_**10**_ with its vacant site, altering the distance from the superatomic core and thereby resulting in a prominent solvatochromic shift at RT.

In summary, this study presents a unique assembly approach employing superatomic Ag NCs, specifically [Ag_10_{S_2_P(O^*i*^Pr)_2_}_8_], as building blocks. The surface characteristics of the **Ag_10_** NC reveal an accessible binding site on the Ag_ext_ atom, enabling the attachment of organic linkers and yielding the formation of [Ag_10_{S_2_P(O^*i*^Pr)_2_}_8_]_2_(μ-4,4′-bpy). Notably, the solvent-dependent UV–vis absorption and emission spectra underscore the substantial influence of the solvent polarity. This research not only introduces innovative approaches to designing supramolecular architectures utilizing superatomic building blocks but also opens a novel avenue for manipulating the photophysical properties of atomically precise Ag NCs. The findings highlight the resilience of superatomic electronic properties, showcasing their capacity for fine-tuning in an atypical way. Further investigations will be warranted to deepen our understanding of the underlying factors governing the successful formation of the targeted assembled architecture.

## Supporting Information Available

Synthesis, NMR spectroscopy, and photophysical data (PDF)
