# Supplementary material for: A Silver Nanocluster Assembled by a Superatomic Building Unit
Source: Inorg Chem. 2024 Mar 12;63(12):5320–4. doi: 10.1021/acs.inorgchem.4c00139 (PMC10966729; doi:10.1021/acs.inorgchem.4c00139)
Supplement: Supplementary file 1 — ic4c00139_si_001.pdf [file ic4c00139_si_001.pdf]

Supporting information

A Silver Nanocluster Assembled by A Superatomic Building Unit

*Wei-Jung Yen,[a] Jian-Hong Liao,[a] Tzu-Hao Chiu,[a] Yuh-Sheng Wen,[b] and C. W. Liu [a,]**

[a] *Department of Chemistry, National Dong Hwa University, Hualien 97401, Taiwan (Republic of China). E-mail: chenwei@ems.ndhu.edu.tw*

[b] *Institute of Chemistry, Academia Sinica, Taipei 11528, Taiwan (Republic of China)*

Experimental Section

All chemicals used as received were purchased from commercial sources. Solvents were purified following standard protocols.[S1] All reactions were performed in oven-dried Schlenk glassware using standard inert atmosphere techniques. All reactions were carried out under N_2 atmosphere by using standard Schlenk techniques. $Ag_{10}\{S_2P(O^iPr)_2\}_8$ was prepared by procedures reported earlier in literature.[S2] The ^1H and $^{31}P\{^1H\}$ NMR spectra were recorded on a Bruker Avance II 400 MHz NMR spectrometer, operating at 400.13 MHz for ^1H, and 161.98 MHz for ^{31}P. The chemical shifts (δ) and coupling constants (J) are reported in ppm and Hz, respectively. X-ray diffraction data were collected on a Bruker APEX II CCD diffractometer. UV-visible absorption spectra were measured on a Perkin Elmer Lambda 750 spectrophotometer using quartz cells with path length of 1 cm. The emission, lifetime, and quantum yield spectra were recorded on an Edinburgh FLS920 fluorescence spectrometer. Quantum yield was determined by a comparative method. Relative values are calculated by using $[Ru(bpy)_3]^{2+}$ as the standard sample. The elemental analysis (C, H, N, S content) of the sample was determined by Elementar UNICUBE elemental analyzer.

Synthesis of $[Ag_{10}\{S_2P(O^iPr)_2\}_8]_2$(-μ-4,4'-bpy), Ag_{10}bpy

$[Ag_{10}\{S_2P(O^iPr)_2\}_8]$ (0.0041 g, 0.0147 mmol) and 4,4'-bipyridine (0.0022 g, 0.147 mmol) were mixed in MeOH/MeCN (1:1) solvent. The solution was concentrated by the evaporator and kept in dark for crystallization for one week to obtain the single crystals of **2**. Yield: 0.0036 g (85.1%, based on Ag). $^{31}P\{^1H\}$ NMR (161.98 MHz, CD_2Cl_2, δ, ppm, 293K): 103.2 (S_2P), 0.65 (sept, $^1J_{PAg}$ = 72 Hz, dppm). $^{31}P\{^1H\}$ NMR (161.98 MHz, $CDCl_3$, δ, ppm): 103.3, 104.4, 105.1. ^1H NMR (400.13 MHz, $CDCl_3$, δ, ppm): 1.35 (d, $^3J_{HH}$ = 7 Hz, 192H, CH_3), 4.87 (br, 32H, CH), 7.55 (br, 4H, bpy), 8.77 (br, 4H, bpy). UV–Vis [λ in nm (ε in M^{-1} cm^{-1})]: 347 (17,100), 389 (12,700), 516 (8,800). Anal. Calcd. for $C_{106}H_{232}Ag_{20}N_2O_{32}P_{16}S_{32}$ ·(CH_3CN): C, 22.49; H, 4.11; N, 0.73; S, 17.79 %. Found: C, 23.72; H, 4.04; N, 0.63; S, 17.70 %.

X-ray Crystallography

Single crystals suitable for X-ray diffraction analysis of **Ag_{10}bpy** were obtained by slow evaporation of methanol solution at ambient temperature. Crystals were mounted on the tip of a glass fiber with paratone oil. Data were collected on a Bruker APEX II CCD diffractometer using graphite monochromated Mo Kα radiation (λ = 0.71073 A) at 100 K. Absorption corrections for area detector were performed with SADABS,[S3] and the integration of raw data frame was performed with SAINT.[S4] The structures were solved by direct methods and refined by least-squares against F^2 using the SHELXL-2018/3

package,[S5] incorporated in SHELXTL/PC V6.14.[S6] All non-hydrogen atoms were refined anisotropically. CCDC no. 2287342 (**Ag_{10}bpy**) contains the supplementary crystallographic data in this article. These data can be obtained free of charge from The Cambridge Crystallographic Data Centre via www.ccdc.cam.ac.uk/data_request/cif.

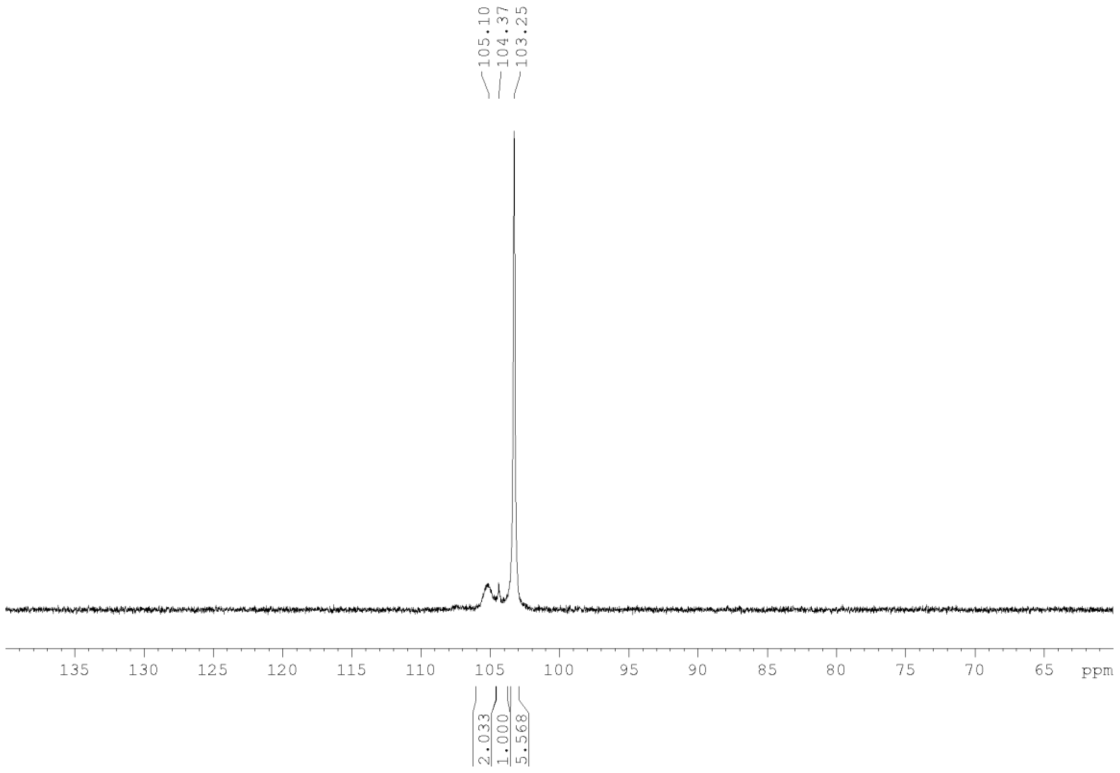

Figure S1. The $^{31}P\{^{1}H\}$ NMR spectrum of **Ag_{10}bpy** (in $CDCl_3$).

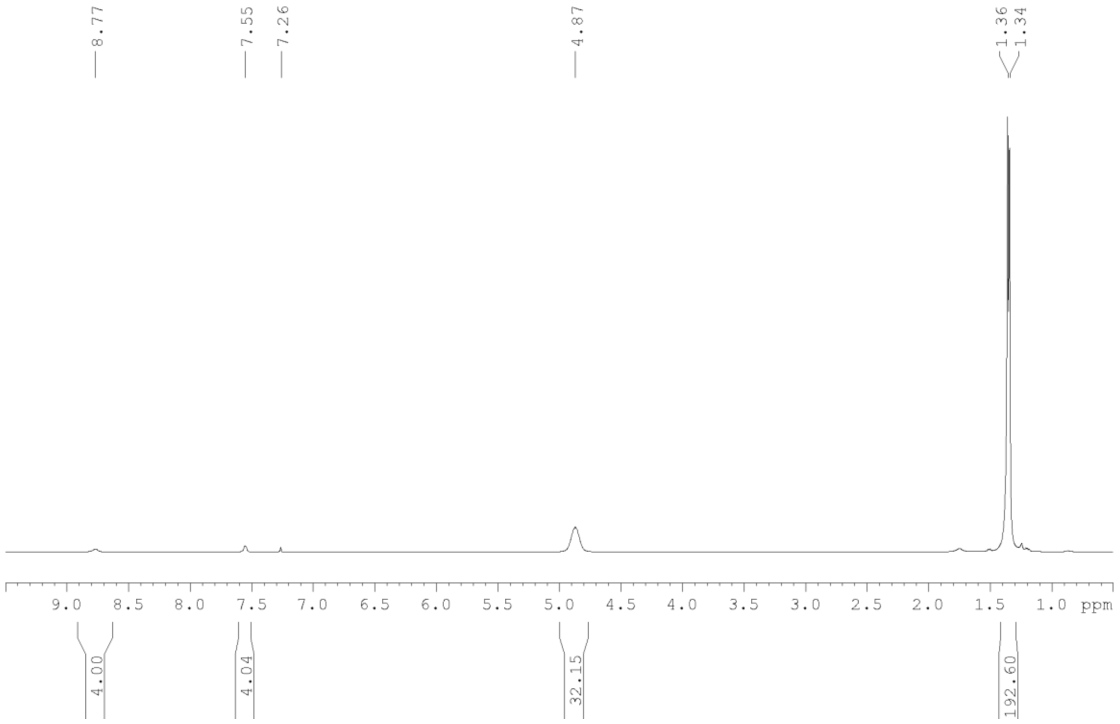

Figure S2. The ^{1}H NMR spectrum of **Ag_{10}bpy** (in $CDCl_3$).

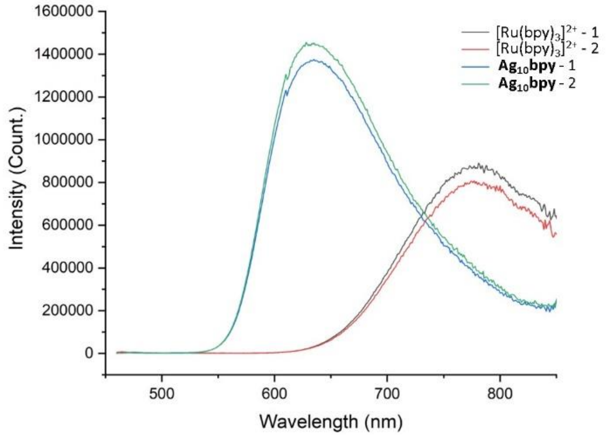

	Solvent	Excite Absorption (A)	Fluorescent area (F)	F/A (*10^8)	F/A Average (*10^8)	F/A Error (%)	QY (Φ_f)	Integral range(nm)
$[Ru(bpy)_3]^{2+}$	H_2O	0.0976	2.19E+08	22.46	22.66	0.90	0.040	460-850
$[Ru(bpy)_3]^{2+}$	H_2O	0.0909	2.08E+08	22.86				
**Ag_{10}bpy**	2Me-THF	0.0914	1.10E+08	12.04	11.63	3.53	0.023	460-850
**Ag_{10}bpy**	2Me-THF	0.1073	1.20E+08	11.22				

Figure S3. The PLQY of **Ag_{10}bpy**.

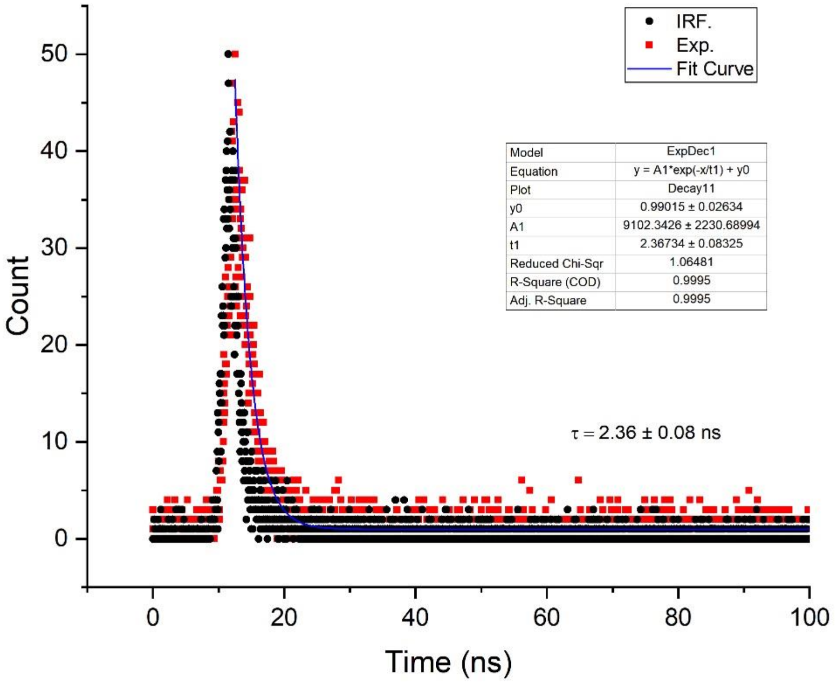

Figure S4. The photoluminescence decay curve of **Ag_{10}bpy** in 2-MeTHF at RT.

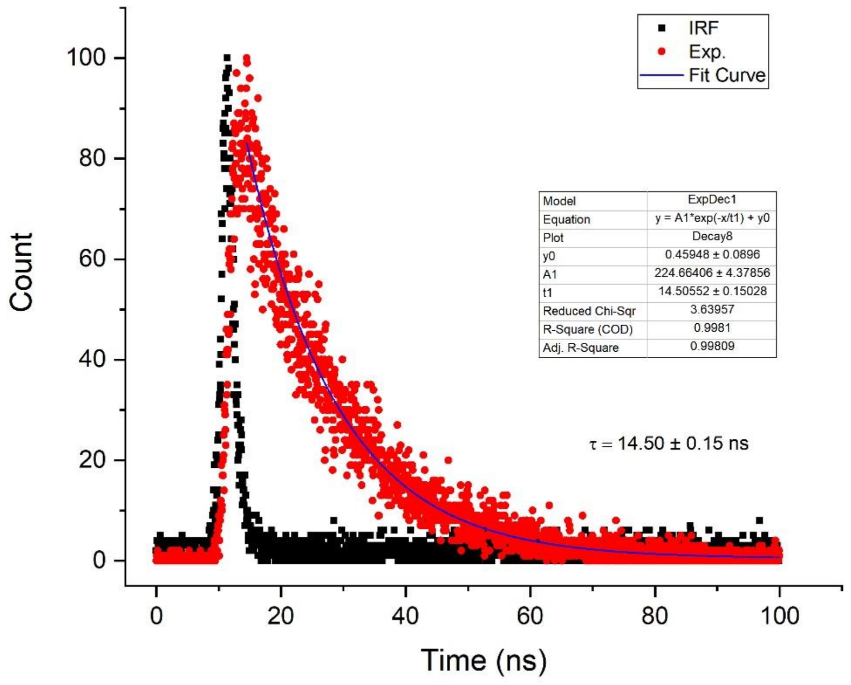

Figure S5. The photoluminescence decay curve of **Ag$_{10}$bpy** in 2-MeTHF at 77K.

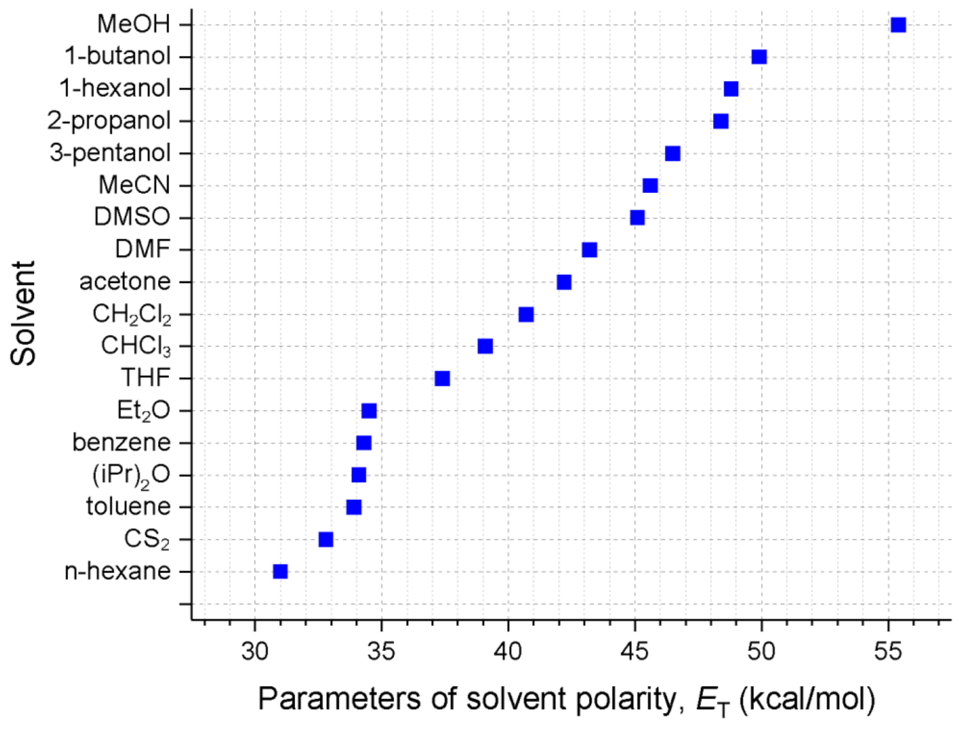

Figure S6. Selected solvent polarity chart.

Table S1. Selected X-ray crystallographic data of **Ag_{10}bpy**.

Compound	**1**
CCDC no.	2287342
Chemical formula	$C_{106}H_{232}Ag_{20}N_2O_{32}P_{16}S_{32}$
Formula weight	5725.76
Wavelength, Å	0.71073
Crystal System	Triclinic
Space group	$P\bar{1}$
a, Å	15.9584(6)
b, Å	26.4546(11)
c, Å	27.7795(10)
α, deg.	64.9002(15)
β, deg.	77.9391(17)
γ, deg.	83.5651(19)
V, $Å^3$	10382.2(7)
Z	2
Temperature, K	296(2)
ρ_{calcd}, g/cm^3	1.832
μ, mm^{-1}	2.335
θ_{max}, deg.	25.00
Completeness, %	99.9
Reflection collected / unique	496869 / 36538 [R(int) = 0.1843]
Restraints / parameters	1079 / 2039
$^a R1$, $^b wR2$ [$I > 2\sigma(I)$]	0.0481, 0.0837
$^a R1$, $^b wR2$ (all data)	0.1153, 0.1084
GOF	1.032
Largest diff. peak and hole, e/$Å^3$	1.588 and -0.866

$^a R1 = \Sigma \mid \mid F_o \mid - \mid Fc \mid \mid / \Sigma \mid F_o \mid$. $^b wR2 = \{\Sigma[w(F_o^2 - F_c^2)^2]/\Sigma[w(F_o^2)^2]\}^{1/2}$.

Table S2. Selected bond lengths (Å) in **Ag$_{10}$bpy**.

Compound	Td Edge Yellow	Td Edge Green	Td Edge Cyan	Td Edge Magenta	Td Height Cyan	S-Ag	N-Ag
cluster **I**	2.7981(9)<br>2.8711(9)<br>2.8813(9)<br>2.8980(9)<br>2.9028(8)<br>2.9487(9)<br>avg. 2.8833(9)<br>**Td Height Yellow**<br>2.334<br>2.354<br>2.359<br>2.388<br>avg. 2.359	2.8284(9)<br>2.8711(9)<br>2.8952(10)<br>2.8980(9)<br>2.9028(8)<br>2.9542(9)<br>avg. 2.8916(9)<br>**Td Height Green**<br>2.323<br>2.338<br>2.353<br>2.391<br>avg. 2.351	2.8711(9)<br>2.8952(10)<br>2.9542(9)<br>3.0929(9)<br>3.0968(10)<br>3.1349(10)<br>avg. 3.0075(10)<br>2.8369(9)<br>2.8927(9)<br>2.9494(9)<br>3.0478(9)<br>3.0772(10)<br>3.1149(9)<br>avg. 2.9865(9)<br>2.7938(9)<br>2.8553(8)<br>2.9531(9)<br>2.9544(10)<br>3.0818(10)<br>3.1199(10)<br>avg. 2.9597(9)<br>2.8515(9)<br>2.8540(9)<br>2.8553(8)<br>2.9494(9)<br>2.9513(9)<br>3.0322(10)<br>avg. 2.9156(9)<br>avg. 2.9567(9) [1]	3.0195(9)<br>3.0388(10)<br>3.0755(9)<br>3.2280(10)<br>3.2946(9)<br>3.3519(11)<br>avg. 3.1681(10)<br>**Td Height Magenta**<br>2.377<br>2.568 [2]<br>2.638<br>2.659<br>avg. 2.561	2.355<br>2.373<br>2.418<br>2.532<br>avg. 2.420<br>2.381<br>2.397<br>2.409<br>2.615<br>avg. 2.451<br>2.340<br>2.341<br>2.411<br>2.485<br>avg. 2.394<br>2.319<br>2.376<br>2.404<br>2.433<br>avg. 2.383<br>avg. 2.412 [3]	2.457(3)<br>2.458(3)<br>2.494(2)<br>2.507(3)<br>2.527(2)<br>2.530(2)<br>2.561(2)<br>2.564(2)<br>2.566(2)<br>2.568(2)<br>2.576(2)<br>2.578(3)<br>2.581(3)<br>2.604(2)<br>2.608(2)<br>2.611(2)<br>2.614(3)<br>2.615(2)<br>2.632(2)<br>2.635(2)<br>2.669(2)<br>2.673(2)<br>2.677(2)<br>2.704(2)<br>2.712(2)<br>2.732(2)<br>2.751(2)<br>2.969(2)<br>avg. 2.613(2)	2.406(7)
cluster **II**	2.8369(9)<br>2.8540(9)<br>2.8553(8)<br>2.8734(9)<br>2.8927(9)<br>2.9494(9)<br>avg. 2.8932(9)<br>**Td Height Yellow**<br>2.287<br>2.351<br>2.365<br>2.385<br>avg. 2.347	2.7938(9)<br>2.8553(8)<br>2.8734(9)<br>2.8927(9)<br>2.8967(9)<br>2.9531(9)<br>avg. 2.8775(9)<br>**Td Height Green**<br>2.322<br>2.333<br>2.351<br>2.383<br>avg. 2.347	2.8927(9)<br>2.8967(9)<br>2.9531(9)<br>2.9574(9)<br>3.0140(10)<br>3.0752(10)<br>avg. 2.9649(9)<br>2.8369(9)<br>2.8927(9)<br>2.9494(9)<br>3.0478(9)<br>3.0772(10)<br>3.1149(9)<br>avg. 2.9865(9)<br>2.7938(9)<br>2.8553(8)<br>2.9531(9)<br>2.9544(10)<br>3.0818(10)<br>3.1199(10)<br>avg. 2.9597(9)<br>2.8515(9)<br>2.8540(9)<br>2.8553(8)<br>2.9494(9)<br>2.9513(9)<br>3.0322(10)<br>avg. 2.9156(9)<br>avg. 2.9567(9) [1]	3.0322(10)<br>3.0410(9)<br>3.0818(10)<br>3.2370(9)<br>3.3351(10)<br>3.6646(1)<br>avg. 3.229(1)<br>**Td Height Magenta**<br>2.328<br>2.593 [2]<br>2.709<br>2.789<br>avg. 2.605	2.349<br>2.366<br>2.434<br>2.587<br>avg. 2.434<br>2.368<br>2.372<br>2.431<br>2.500<br>avg. 2.418<br>2.324<br>2.368<br>2.380<br>2.419<br>avg. 2.373<br>2.314<br>2.324<br>2.435<br>2.558<br>avg. 2.408<br>avg. 2.408 [3]	2.454(2)<br>2.460(2)<br>2.481(3)<br>2.494(2)<br>2.514(2)<br>2.530(2)<br>2.545(2)<br>2.548(3)<br>2.556(2)<br>2.556(2)<br>2.576(2)<br>2.581(2)<br>2.588(2)<br>2.598(2)<br>2.600(2)<br>2.610(2)<br>2.619(2)<br>2.645(2)<br>2.652(2)<br>2.660(2)<br>2.664(3)<br>2.669(2)<br>2.669(2)<br>2.670(2)<br>2.720(2)<br>2.744(2)<br>2.793(2)<br>2.951(2)<br>avg. 2.612(2)	2.395(7)

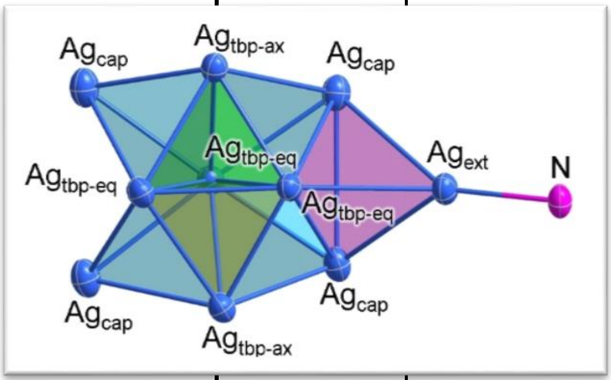

Colored background represents the average bond distances. [1] The average bond length of all cyan tetrahedrons. [2] Distances from Ag$_{ext}$ to ΔAg$_{tbp-eq}$-Ag$_{cap}$—Ag$_{cap}$. [3] The average height of all cyan tetrahedrons.

Table S3. The photophysical data of **Ag_{10}bpy**.

Comp.	State	Absorbance λ_{abs} (nm), ε (M^{-1} cm^{-1})	Excitation λ_{ex} (nm)	Emission λ_{em} (nm)	Lifetime (ns)	Quantum Yield (%)	k_r (s^{-1})[a]	k_{nr} (s^{-1})[b]
**Ag_{10}bpy**	2-MeTHF, 298K	347 (17,100), 389 (21,700), 516 (8,800)	330, 405, 521	749	2.36	2.3	9.75×10^6	4.14×10^8
	2-MeTHF, 77K		333, 384, 514	686	14.5			

[a] k_r: radiative decay. [b] k_{nr}: nonradiative decay.

$$\tau = \frac{1}{k_r + k_{nr}} \qquad QY = \frac{k_r}{k_r + k_{nr}}$$

References

S1. Perrin, D. D.; Armarego, W. L. F. *Purification of laboratory chemicals*, 3rd ed.; Pergamon Press: Oxford, 1988.

S2. Zhong, Y.-J.; Liao, J.-H.; Chiu, T.-H.; Kahlal, S.; Lin, C.-J.; Saillard, J.-Y.; Liu, C. W. *Angew. Chem. Int. Ed.* **2021**, *60*, 12712–12716.

S3. SADABS version 2014−11.0, Bruker Area Detector Absorption Corrections; Bruker AXS, Inc.: Madison, WI, 2014.

S4. SAINT V8.30A, Software for the CCD Detector System; Bruker Analytical: Madison, WI, 2012.

S5. Sheldrick, G. M. A short history of SHELX. *Acta Cryst. A* **2008**, *64*, 112.

S6. SHELXTL Version 6.14; Bruker AXS, Inc.: Madison, WI, 2003.
